# Metal Fused Filament Fabrication (MF^3^) of CoCrMo Feedstocks

**DOI:** 10.3390/ma19214558

**Published:** 2026-10-29

**Authors:** Thomas Hanemann, Steffen Antusch, Dorit Nötzel, Tom Reimold, Iwiza Tesari, Ralf Eickhoff

**Affiliations:** 1 Institute for Applied Materials, Karlsruhe Institute of Technology, Hermann-von-Helmholtz-Platz 1, 76344 Eggenstein-Leopoldshafen, Germany; 2 Department of Microsystems Engineering, University of Freiburg, Georges-Köhler-Allee 102, 79110 Freiburg, Germany

**Keywords:** CoCrMo, MF^3^, fused filament fabrication, PEG/PMMA-based binder

## Abstract

**Highlights:**

**What are the main findings?**

- It was possible, to develop new highly filled feedstock systems with CoCrMo as metal filler successfully.
- The principal feasibility of metal fused filament fabrication of small and mid-sized parts was demonstrated.
- Metal parts with an acceptable sinter density after debinding and sintering were obtained.

**What are the implications of the main findings?**

- The MF^3^ process chain for the realization of small and mid-sized parts has been established.

**Abstract:**

CoCrMo is one of the most important metal alloys for the fabrication of medical implants. The classic implant fabrication method is casting; in recent years, selective laser or electron beam melting as additive manufacturing methods have been established. Both methods mainly deliver parts with an anisotropic texture, which may not be advantageous for a lot of applications. In addition, both techniques are cost-intensive. In this work, the feasibility of the process chain of metal fused filament fabrication (MF^3^), covering the individual steps of compounding, printing, debinding and sintering, was investigated. Of particular importance is the feedstock composition consisting of suitable CoCrMo powder with a solid load of 60 Vol%, polyethylene glycol (PEG) and polymethylmethacrylate (PMMA) as binder components. Furthermore, acetyltributylcitrate (ATBC) was used as the plasticizer, necessary for a flexible filament, which can be wound on a spool. The influence of the ATBC content on compounding, feedstock melt viscosity and printing behavior was investigated. Samples of different sizes, like cuboids and tensile test specimens were debound and sintered to CoCrMo parts. A significant influence from the final sinter temperature on the part’s appearance and density was observed. Sintering at 1350 °C resulted in parts with a maximum relative density of almost 95% while retaining the shape of the green bodies. The slight increase to 1400 °C caused a partial sample melting with structural deformations visible at the edges, corners and at the surface. However, a density better than 99% could be reached. The measured ultimate tensile strength is lower than the reference value achieved with investment casting, but the elongation at break is equal or even higher. Nevertheless, the principal feasibility of the MF^3^ process chain for the realization of CoCrMo components could be demonstrated successfully.

## 1. Introduction

In Germany and worldwide, population aging and the related social and economic consequences are key challenges for the next couple of years [1,2]. The number of people in Germany with an age of 70 years or higher rose between 1990 and 2022 from 8 to 14 million [1]. This trend is accompanied by an increase in the larger number of people reaching a higher life expectancy. It is expected that worldwide the percentage of people older than 65 will increase from 9.7% to 16.4%. Focusing on Europe and North America, as well as Eastern and South-Eastern Asia, the percentage rises from 13–19% (2022) to around 26%. Consequently, there will be a significant need for medical implants, like artificial hip or knee joints, due to an expectable rise in age related injuries [3]. In addition, here reported for Germany, the number of older humans, who are active in sports at least once per week, is quite high [4], which may cause an increasing demand for implants according to sport-related accidents or joint wear like arthrosis. Another important factor is the increasing surplus weight in the western world [5,6].

Three different metal materials are established in biomedical applications focusing on hard tissue: titanium alloys, cobalt alloys and nickel-free stainless steels [7]. A classic fabrication method of Ti-6Al-4V (Ti64)-based implants is forging, as defined in [8]. Nowadays, additive manufacturing methods, like selective laser melting (SLM) and electron beam melting (EBM), followed by post-treatment like further densification, homogenization of the microstructure, and surface treatment for enhanced biocompatibility, gaining more importance due to the principal possibility, to manufacture patient-specific and customized implants [9,10]. In contrast, implants made from CoCrMo alloys, as defined in ASTM F75, were typically fabricated by different casting methods followed by various postprocessing techniques like further densification, milling, turning, sanding and polishing [11,12,13]. As an alternative, forged or wrought alloys are used [14,15].

Additive manufacturing methods, like SLM and EBM, are also of rising relevance [16,17,18,19]. Wang et al. investigated the impact of SLM parameters on the properties of Co-Cr alloys [20]. Guoqing and coworkers described an additional heat treatment of CoCrMn according to ASTM F1377 alloy after fabrication by SLM and the resulting mechanical property changes [21]. A similar investigation plus adding hot isostatic pressing was undertaken by Cornacchia et al. on for SLM designated commercial Co28Cr6Mo (ASTM F75) [22]. The impact of different sinter conditions on the resulting CoCrMo properties were investigated by Lemus-Ruiz et al. [23]. They varied the sinter temperature between 1300 and 1375 °C and measured microhardness, shrinkage and microstructure. From the obtained data, they recommend sintering the alloy between 1350 and 1375 °C. Unfortunately, they did not mention the exact composition of the commercial CoCrMo alloy used [23]. As with Ti64, the use of additive manufacturing methods allows for individual customized implant sizes enabling acceleration of wound healing and mobilization. Metal injection molding (MIM) is an accepted fabrication method for medical implants or surgical tools [24,25] but does not allow the fabrication of customized products. In recent years fused filament fabrication (FFF) as part of the material extrusion (MEX) techniques, originally designed for polymers, was further developed for the realization of metal (denoted as MF^3^: metal fused filament fabrication) and ceramic (denoted as CF^3^: ceramic fused filament fabrication) parts. As in MIM, MF^3^ involves an extensive process chain with the individual steps of feedstock formation (compounding), shaping and thermal postprocessing such as debinding and sintering to obtain final dense metal parts. The key element is the feedstock composition, consisting of the solid filler (metal or ceramic) and the binder system, usually consisting of at least two polymers and various additives, e.g., for improved filler wetting, feedstock viscosity adjustment and plasticizing [26].

Common binder systems for MIM consist of a low molecular mass polymer, like wax or PEG (polyethylene glycol), a high molecular mass polymer, like LDPE or HDPE (low- or high-density polyethylene), PMMA (polymethylmethacrylate) and PVB (polyvinylbutyral), and a surfactant, like SA (stearic acid) and other related fatty acids [27,28]. Typically, the low molecular mass polymer is removed in a liquid pre-debinding step using appropriate solvents like hexane (wax) and water (PEG). The high molecular mass polymer is thermally removed prior to sintering.

Only a small amount of research describes applying MEX or related methods for the additive manufacturing of CoCrMo parts. Pellegrini and coworkers used metal binder jetting as a printing technique [29]. They applied a Co28Cr6Mo ASTM F75 alloy in a commercial binder jetting printer from Desktop Metal Inc. Thermal postprocessing like debinding and sintering were performed using predefined parameters from the furnace supplier. The maximum temperature was 1325 °C for 4 h. The achieved sinter densities were in the range between 93.7 and 97.5% th. with an average value of 94.9 ± 1.4% th. [29]. Garcia de la Cruz et al. applied the same ASTM F75 alloy in combination with granule-based MEX, also known as fused granulate fabrication (FGF) or pellet extrusion [30]. They used a paraffin wax/HDPE (high density polyethylene) binder without further additives. The solid load varied between 50 and 60 Vol%. The green-body densities were in the range between 94 and 96%, the best sinter density achieved was 98.5% th. after sintering at 1375 °C under vacuum for 2 h [30].

In recent years, MF^3^ was successfully used for the realization of dense Ti64 implant structures like hip endoprosthesis or for dental applications [31,32]. The aim of this work is to extend the knowledge gained so far to CoCrMo F75 alloy as a second important implant material and to show the principal feasibility of the MF^3^ process chain in addition to the described FGF printing method [30]. From an economic point of view, the different MEX methods, like the reported FGF or the MF^3^, have a great potential to lower the costs of, e.g., customized printed implants. Prior to this, the TRL level must be increased significantly, which should be realistic within the next few years.

## 2. Materials and Methods

### 2.1. Material Selection

**Figure 1:**
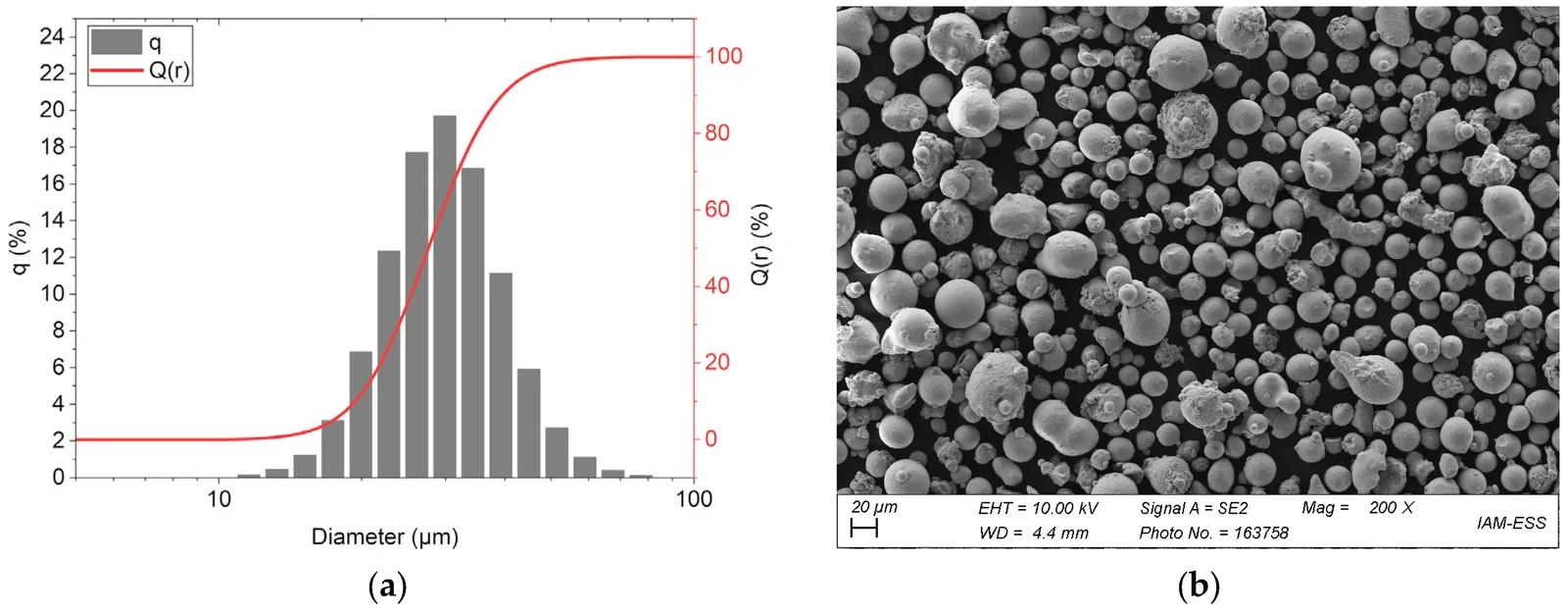
CoCrMo particles: (**a**) measured particle size distribution; (**b**) SEM image showing an almost spherical morphology.

A commercial CoCrMo alloy ASTM F75 [11] from Carpenter Additive (Philadelphia, PA, USA) was selected as metal powder [33]. According to the vendor, the particle size range is between 15 and 45 µm, which was verified by our own measurements (LA-950, Retsch Technology, Haan, Germany) (Table 1). In addition, the specific surface area according to the BET method (Micromeritics Instrument Corporation, Norcross, GA, USA) and powder density via helium pycnometry (AccuPyc II 1340 V2.01, Micromeritics, Norcross, GA, USA) were measured for comprehensive characterization. Figure 1 shows the entire particle size distribution and the mostly spherical morphology of the metal particles using SEM (Supra 55, Zeiss, Oberkochen, Germany).

In continuation of previous work dealing with the MF^3^ feedstock development [31,32], a binder system consisting of PMMA as backbone polymer, responsible for a certain mechanical stability of the filament and the printed green bodies, PEG as the water-soluble main binder, responsible for a low melt viscosity as well as surfactant and acetyltributylcitrate (ATBC) as plasticizer, were investigated. Table 2 lists all the binder components used in the new feedstock systems and their respective functions. As in previous MF^3^ feedstocks, the solid load was set to 60 Vol%, which is a reliable compromise between good MF^3^ printability and acceptable sinter shrinkage. The amount of additive has a particular impact on processing; hence, three different ATBC concentrations were researched (Table 3). The increasing ATBC concentration was compensated by a reduction in the amount of PEG 8000.

### 2.2. Compounding and Rheological Characterization

The preparation of the new feedstock systems was realized applying a torque recording compounder (W50 EHT, Brabender, Duisburg, Germany), which enables an in-line torque recording during mixing, visualizing the compounding progress at a given temperature with proceeding time. The selected compounding parameters were 170 °C and 1 h mixing with a mixing speed of 60 rpm. The volume of the mixing chamber is fixed at 50 cm^3^. Earlier work showed that the addition sequence of the different binder components into the mixing chamber should be retained in all experiments; therefore, initially a small portion of the metal filler was given into the chamber, followed by a complete addition of PMMA, PEG and ATBC and concluded with the remaining metal powder. After feedstock preparation, the mixtures were characterized using a high-pressure capillary rheometer (Rheograph 25, Goettfert GmbH, Buchen, Germany, capillary length 30 mm, capillary diameter 1 mm) at a measuring temperature of 160 °C. The temperature value was taken from the literature [31]. The shear rate range was varied between 1 and 5000 s^−1^. The obtained viscosity data were corrected according to the Weißenberg–Rabinowitsch method [34].

### 2.3. Filament Extrusion

Filaments prepared from the three different feedstocks were extruded by a single-screw filament extruder (Noztek Pro, Noztek, Shoreham-by-Sea, UK) and subsequently wound onto a spool using a filament winder (Winder 1.0, Noztek, Shoreham-by-Sea, UK). The applied nozzle diameter was 3.05 mm.

### 2.4. FFF Printing

A German RepRap x350 pro (German RepRap GmbH, Feldkirchen, Germany) MEX printer was used. The pristine filament extruder was substituted with an extruder from Bondtech (QR, Bondtech AB, Värnamo, Sweden) due to the presence of a more precise dual gear filament drive suitable for soft filaments. The modified printhead was additionally equipped with optional part cooling for a higher resolution of the printed parts and the realization of complex geometries. The printing parameters used can be found in Section 3.3.

### 2.5. Selected Test Structures

**Figure 2:**
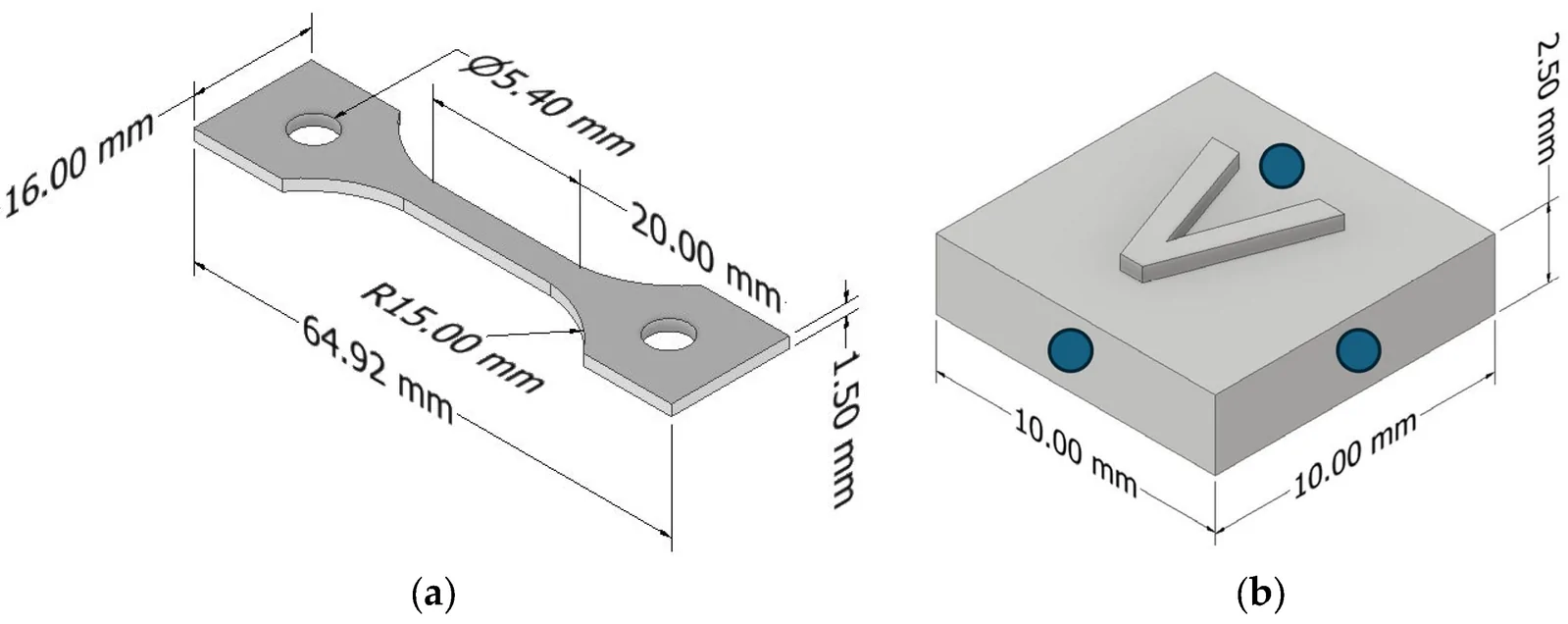
Printed test structures (all dimensions in mm): (**a**) tensile test specimen (sample width in the middle: 4 mm); (**b**) cuboid.

**Figure 3:**
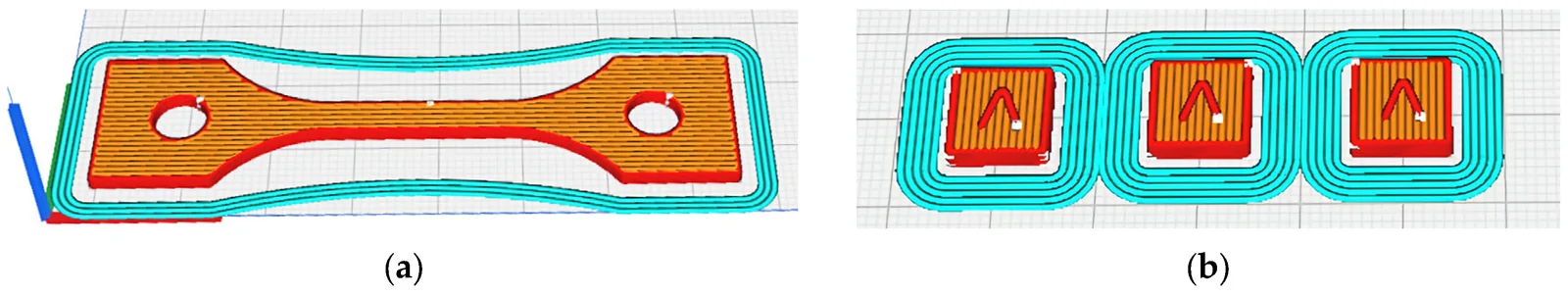
Printing strategies: (**a**) tensile test specimen; (**b**) cuboids.

Two different test specimens for addressing different purposes were selected. Samples for tensile testing were printed with respect to the characterization of mechanical properties (Figure 2a). Cuboids were selected for the estimation of sinter shrinkage (Figure 2b), part density and for metallographic characterization after sintering. The blue dots represent the positions for the x,y,z measurements. The printing strategy was not unique: Whilst in case of the tensile test specimen the printing direction was parallel to the tensile load in all printed layers (Figure 3a), the printing directions were altered by 90° for the cuboids (Figure 3b). In total, 48 cuboids (nozzle diameter 0.8 mm) and 50 tensile testing specimens (30× with 0.8 mm nozzle diameter, 10× 0.6 mm nozzle diameter, 10× 0.4 mm nozzle diameter) were printed. Ultimaker Cura 5.4 was applied as slicer for the generation of the G-code files.

### 2.6. Debinding and Sintering

**Figure 4:**
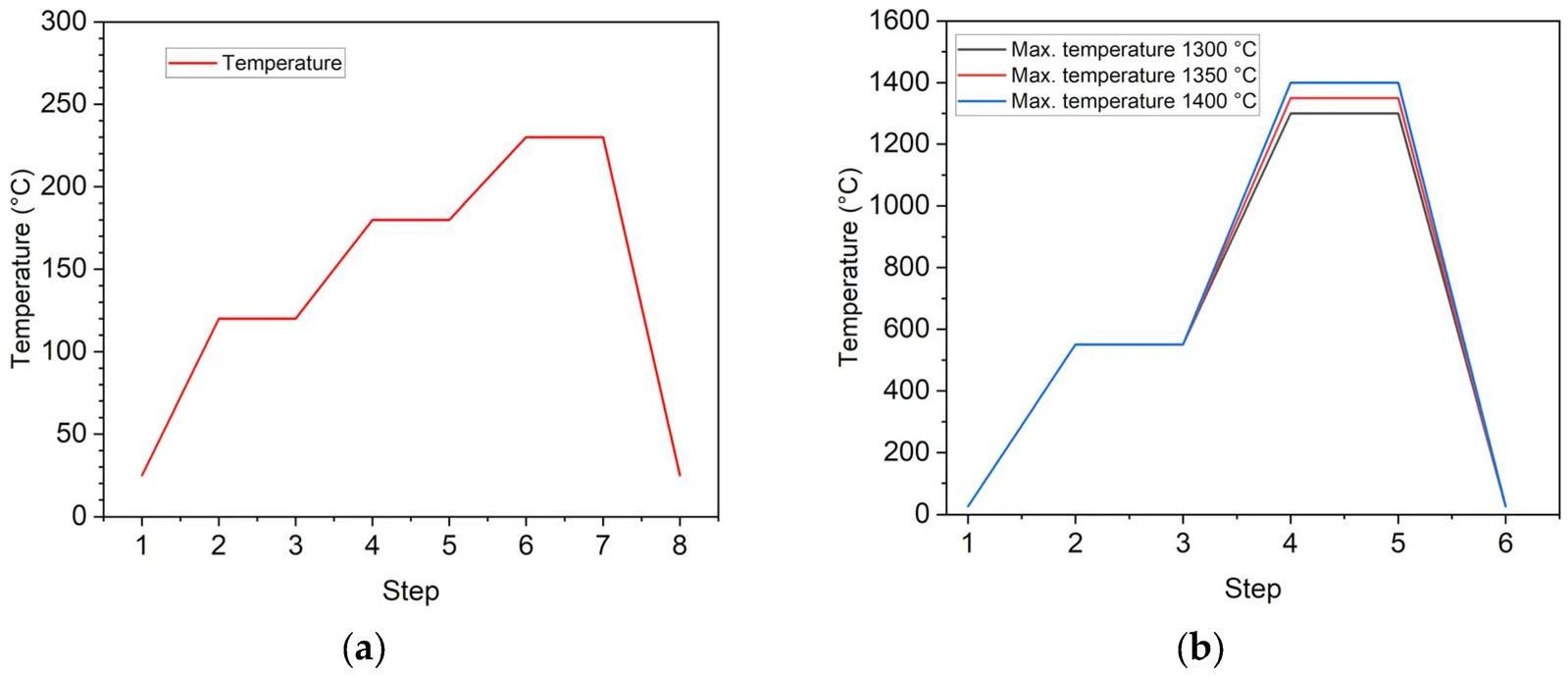
Thermal programs; (**a**) pre-debinding, (**b**) combined debinding and sintering.

The usage of large amounts of ATBC as binder component required a modification in the common debinding procedure—liquid pre-debinding and thermal debinding—by addition of a thermal pre-debinding step. The liquid pre-debinding was performed in water for 48 h (tensile test specimen) or 24 h (cuboids) at 30 °C to remove the PEG completely. The elimination of the ATBC proceeded at 230 °C with very low heating rates in combination with dwell times at certain temperatures (Table 4) using a Carbolite HT6/28 chamber furnace (Carbolite Gero, Neuhausen, Germany). Finally, thermal debinding and sintering under argon atmosphere (metal sinter furnace MUT Advanced Heating GmbH, Jena, Germany) enabled the realization of sintered metal parts. For better process parameter–property evaluation, three different sinter temperatures and two different dwell times at maximum temperature were selected (Table 5). For better visualization, the thermal pre-debinding and the sinter program are presented in Figure 4.

### 2.7. Sintered CoCrMo Part Characterization

All sintered parts were characterized with respect to the achieved sinter density, which was measured according to the Archimedes principle using a YDK01 balance Sartorius (Sartorius, Göttingen, Germany) with water as solvent. The shrinkage of the sintered parts was estimated by a caliper screw VRZ1 (Johannes Heidenheim GmbH, Traunreut, Germany) at certain measuring positions (blue dots in Figure 2b). The cuboids destinated for metallographic investigations were split into parts with a corundum circular saw ATM Brilliant 200 (ATM Qness GmbH, Mammelzen, Germany). The incorporation of the sample in an epoxide resin was performed with an Opal 410 (ATM Qness GmbH, Mammelzen, Germany). After solidification, the surface was sanded (granularity 320 and 800) and prepolished with a diamond suspension (9 μm). Surface finishing was done with Eposil M (colloidal silica, 0.06 μm) at an ATM-sapphire (both ATM Qness GmbH, Mammelzen, Germany). Finally, the samples were etched electrochemically using a carbon electrode with 30% HCl/H_2_O_2_ at 4 V for 20 s. Microstructural studies were performed using a high-resolution field emission scanning electron microscope (SEM) Zeiss Merlin (Carl Zeiss, Jena, Germany). Microscopic images were recorded using a Leica Wild M10 stereomicroscope (Leica Microsystems, Wetzlar, Germany). Nondestructive examination using CT-scans (Phoenix v tome xs, General Electric, Frankfurt, Germany) of selected samples enabled the visualization of inner defects like voids or cracks. The accessible spatial resolution was 15 µm (measuring time: 100 ms; voltage: 10 kV, current: 120 µA).

### 2.8. Mechanical Characterization

Tensile strength and elongation at break were determined by means of tensile tests on a universal testing machine (Instron 1185, Instron GmbH, Darmstadt, Germany). For each variant investigated, five samples were tested. For minimization of bending effects, the specimens were mounted on a fully gimbaled suspension system. The tests were performed under displacement control with a constant crosshead speed of 1 mm/min. The forces acting on the samples during testing were measured with a 10 kN load cell mounted onto the crosshead. The elongation, based on an initial gauge length of 18.8 mm, was recorded with a MTS displacement transducer (MTS Messtechnik Schaffhausen GmbH, Stein am Rhein, Switzerland).

## 3. Results and Discussion

### 3.1. Feedstock Compounding

As listed in Table 3, three feedstocks with different ATBC concentrations at a constant CoCrMo load (60 Vol%) were compounded by a torque-recording mixer-kneader system. Figure 4a shows the proceeding compounding process measured by the torque change with mixing time. As described earlier [35], the general torque evolution can be split into three stages:

1. Filling state: Adding all materials into the mixing chamber in an identical sequence;
2. Mixing state: Start of particle wetting by the surfactant;
3. Stationary state: Equilibrium between surfactant adsorption and desorption.

**Figure 5:**
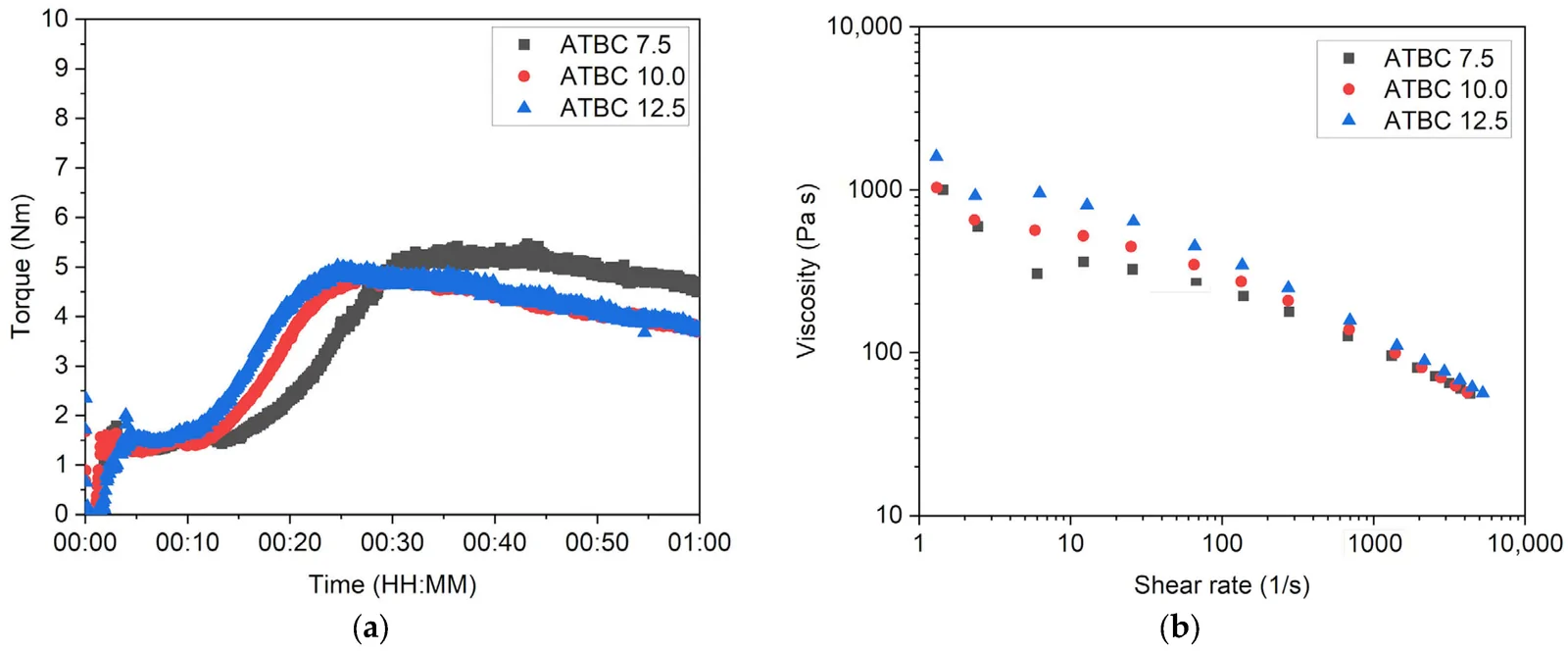
Characterization of the investigated feedstocks: (**a**) compounding progress with time at 170 °C, (**b**) melt viscosity evolution with shear rate at 160 °C.

The investigated feedstocks do not behave in a typical way as shown in [35] (Figure 5a). Normally during the initial filling stage, a maximum torque value can be seen due to an enhanced particle–particle friction prior to the wetting process. During the mixing stage, the torque drops due to particle wetting by the surfactant followed by the stationary state with an almost constant torque. In this case, the torque rises after the filling stage (time from 00:00 to 00:04 HH:MM) by a factor of approximately three, stays for a long period at this maximum value around 5 Nm in the mixing state (00:04 to 00:25–00:35 HH:MM), followed by a slow decay reaching the equilibrium state (00:25–00:35 to 01:00 HH:MM). This unusual behavior has been reported earlier in [36] and can be attributed to the used PMMA type G7E. G7E consists of standard technical pellets with a typical diameter of around 2 mm and a length of 3–4 mm; the average molecular mass M_W_ is around 159,000 g/mol [36]. As polymers possess poor thermal conductivity, the granules were not yet liquified at the end of the filling stage. The melting process occurs slowly and is finished at the maximum torque value around 5 Nm. After that, the wetting process can start, and a homogenous feedstock can be obtained after one hour. A further extension of the compounding time towards two hours delivers only a small additional torque decay and was therefore omitted. The increasing ATBC content, especially from 7.5 to 10 Wt%, causes a reduction in the time needed for reaching the maximum torque value. This can be attributed to a pronounced plasticizing and mixing effect.

### 3.2. Melt Viscosity

Depending on the diameter of the nozzle used, the maximum shear rates are between 50 and 100 1/s during FFF printing. In contrast, in injection molding, shear rates up to 10,000 1/s can be observed, which can be attributed to the huge polymer melt injection speed. Ceramic- or metal-filled feedstocks show a pronounced shear thinning behavior [31], which simplifies injection molding and hampers FFF printing. In Figure 5b, the shear rate-dependent melt viscosity at 160 °C can be seen. At very low shear rates below 10 1/s, the Newtonian plateau can be assumed; at higher shear rates all investigated feedstocks show the typical shear-thinning (pseudoplastic) flow [31]. Interestingly, at shear rates up to 100 1/s, a pronounced impact of the ATBC content on the melt viscosity can be detected: increasing ATBC amounts yield a viscosity increase. This surprising effect can be explained by the fact that increasing ATBC amounts caused a drop in the PEG amount. PEG is responsible for the reduction in viscosity under melt conditions, whilst the PMMA content remains constant. At larger shear rates the resulting viscosities approach each other due to the complete polymer chain de-entanglement.

### 3.3. Filament Extrusion

Table 6 shows for all systems the optimized filament extrusion parameters and the obtained filament diameter. The latter one was measured via a micrometer caliper under ambient conditions. Due to the large amount of ATBC, the filament was flexible at extrusion temperature and could be wound onto a spool.

### 3.4. FFF Printing Process Evaluation

In general, all three investigated feedstocks could be printed via FFF. All relevant printing parameters can be found in Table 7. However, the ATBC 7.5 filament is under ambient conditions very brittle and tends to break at the print head inlet. In contrast, the ATBC 12.5 filament possesses a soft surface and grinding during feeding can be observed. Therefore, in the following ATBC 10.0 was mainly used for further investigations.

**Figure 6:**
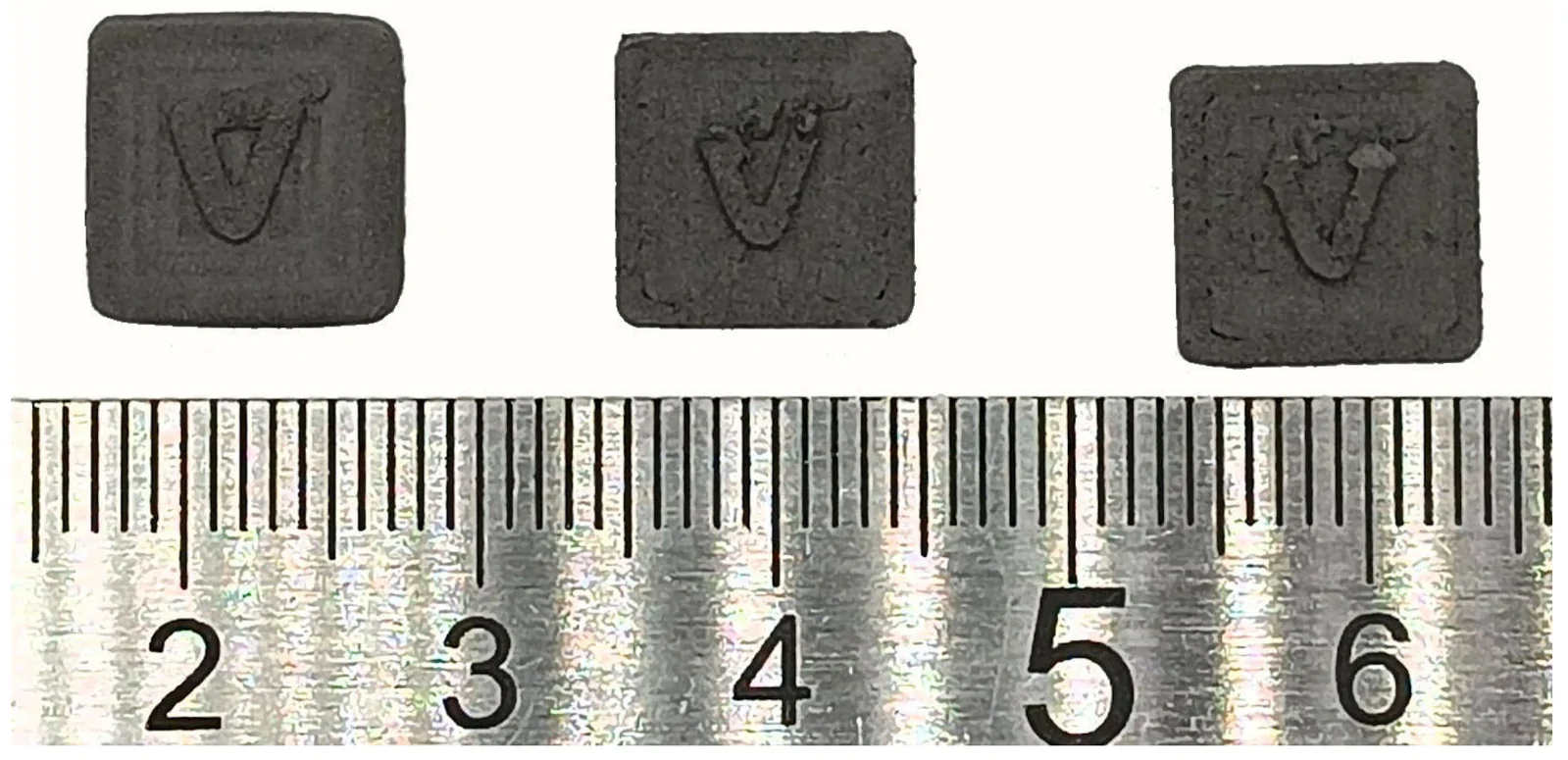
ATBC 10.0 cuboids, printed at different temperatures (left to right: 190 °C, 185 °C, 180 °C).

Exemplarily, Figure 6 shows cuboids out of ATBC 10.0 printed at different temperatures. From the three different samples, it is clearly depicted that the highest applied printing temperature of 190 °C delivered the best part quality. At lower temperatures a huge number of voids, individual strands and pronounced printing defects can be seen. As a result, for further printing the temperature was set to 190 °C.

**Figure 7:**
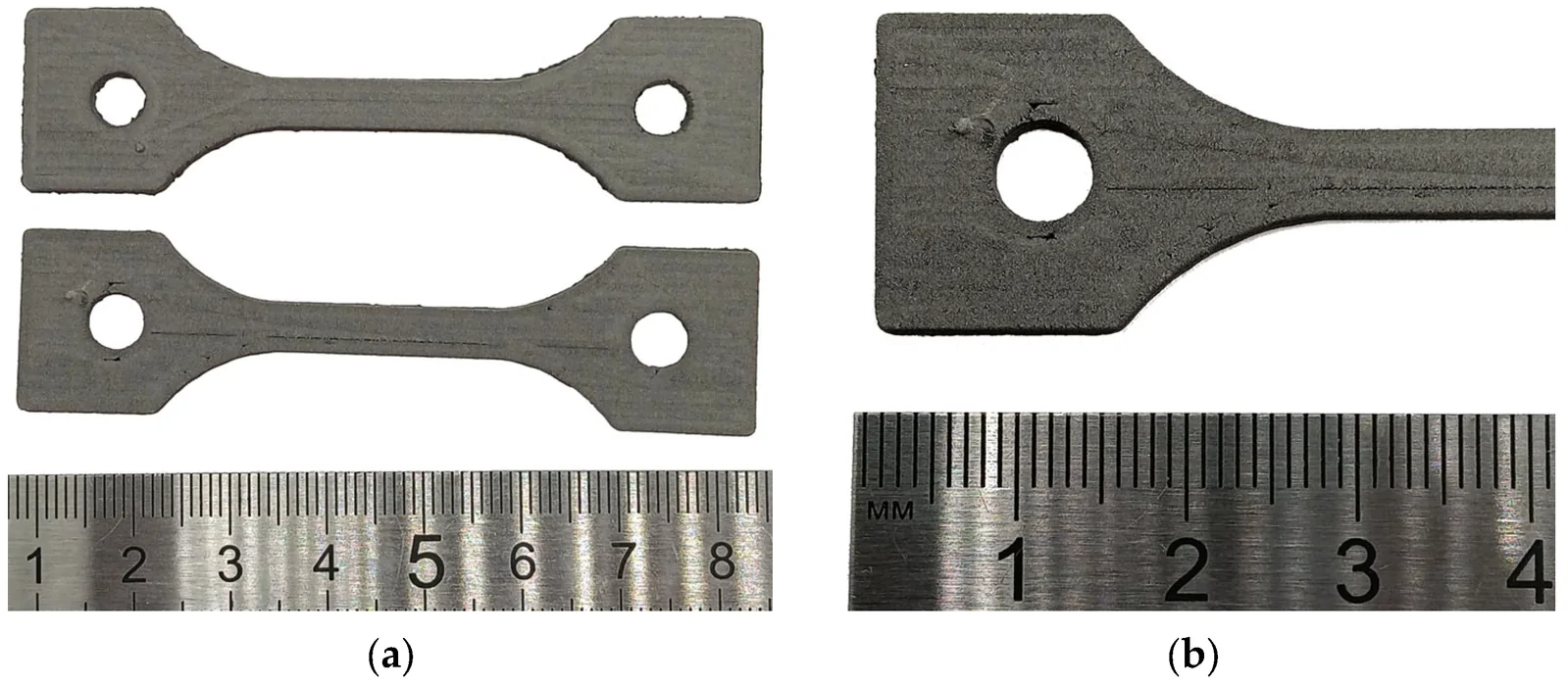
Printed tensile testing bars: (**a**) overlap 0.6 mm (**above**) and 0.4 mm (**below**); (**b**) detailed view of sample with overlap 0.4 mm.

With respect to a quantitative evaluation of the print quality, tensile testing bars were printed at 190 °C with a nozzle diameter of 0.8 mm according to the above results. The overlap parameter (see Table 7) was varied between 0.4 mm and 0.6 mm, the resulting parts are depicted in Figure 7. The sample with the lower overlap value of 0.4 mm possesses severe surface defects in contrast to the sample with an overlap of 0.6 mm. Therefore, the latter one was set as fixed parameter for MF^3^ printing. In addition, the feedstock system ATBC 10.0 delivered the best printing results and was therefore used for all further printing experiments.

### 3.5. Debinding

**Figure 8:**
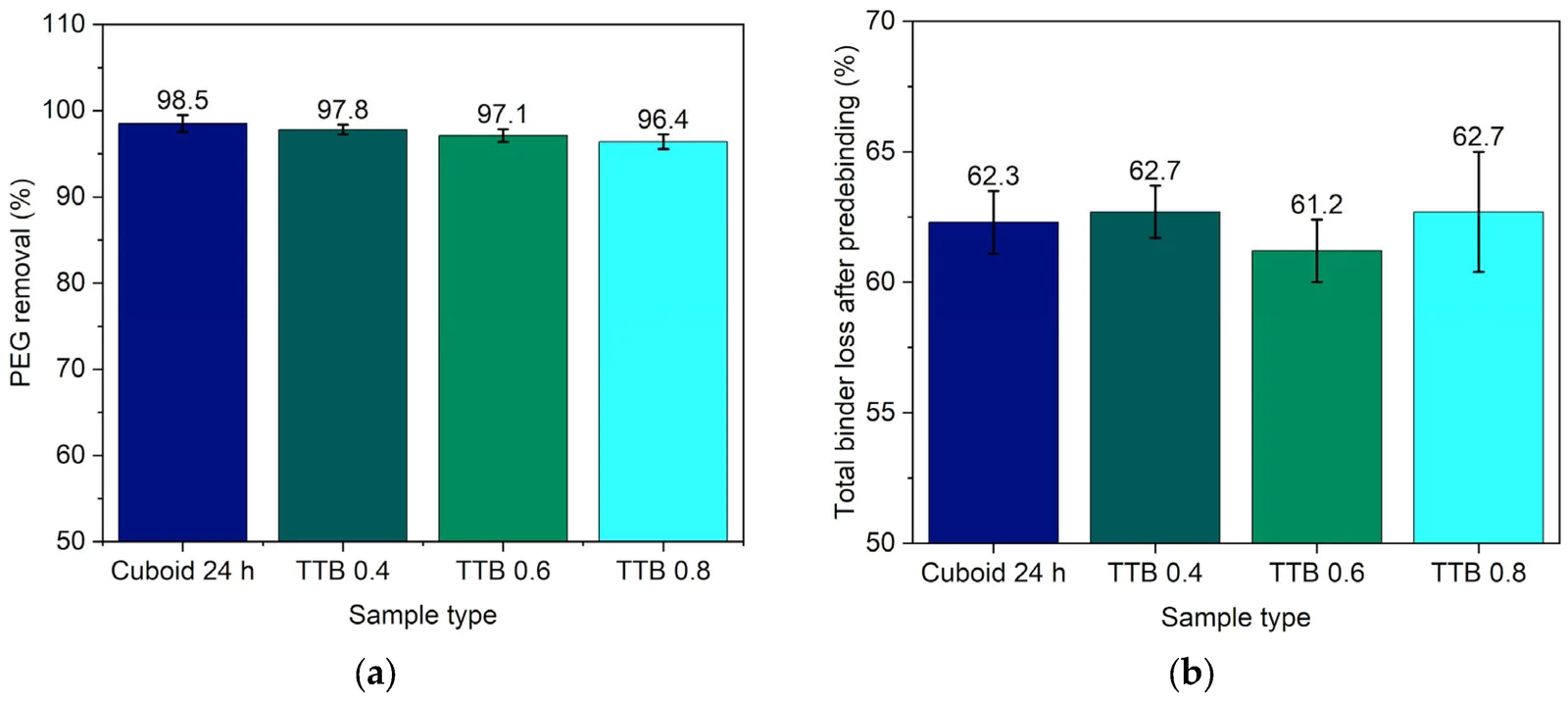
Feedstock pre-debinding: (**a**) relative PEG loss during liquid pre-debinding in water; (**b**) total binder loss after thermal pre-debinding at 230 °C.

As described earlier, only ATBC 10.0 was further considered. In the first debinding step the water soluble PEG8000 was removed by placing the samples in deionized water at 30 °C. This process step is a common method for metal- or ceramic-filled feedstocks according to the pore formation during PEG dissolution, which allows the diffusion of gaseous polymer fragments, water or carbon dioxide, out of the bulk material without damaging the sample part during thermal debinding [31]. To achieve this, an almost complete removal of the PEG is desirable. Depending on the sample size, the storage time in water was not unique. Figure 8a shows the average relative PEG-removal rate of different printed samples (48 cuboids), 10× tensile test bars 0.4 (TTB 0.4), 10× tensile test bars 0.6 (TTB 0.6), 30× tensile test bars 0.8 (TTB 0.8). As a general trend, the smaller the sample the higher the PEG-removal rate due to short diffusion paths. Within the tensile test specimen series, an increasing nozzle diameter lowers the PEG-removal rate slightly. A smaller nozzle diameter means a smaller strand width and a larger number of layers at an identical sample thickness. A larger number of layers is equivalent to a larger number of welding lines which seem to support the PEG’s diffusion process in water. However, the liquid pre-debinding did not cause any visible surface defects. Previous investigations showed that the usage of larger amounts of ATBC required an additional thermal debinding step prior to sintering to avoid a part damage like surface blistering [35]. ATBC possesses a boiling temperature of 331 °C under ambient pressure but exhibits a remarkable vapor pressure even at low temperatures [37]. Own thermogravimetric investigations on ATBC showed that, starting around 200 °C, a pronounced sample weight loss due to ATBC evaporation can be detected, which finishes around 310 °C. Figure 8b shows the measured total binder loss after liquid pre-debinding (PEG) and thermal pre-debinding (ATBC). The data for the cuboid and the tensile testing bars are very close together with values around 62%.

### 3.6. Sintered Part Characterization

#### 3.6.1. Density

**Figure 9:**
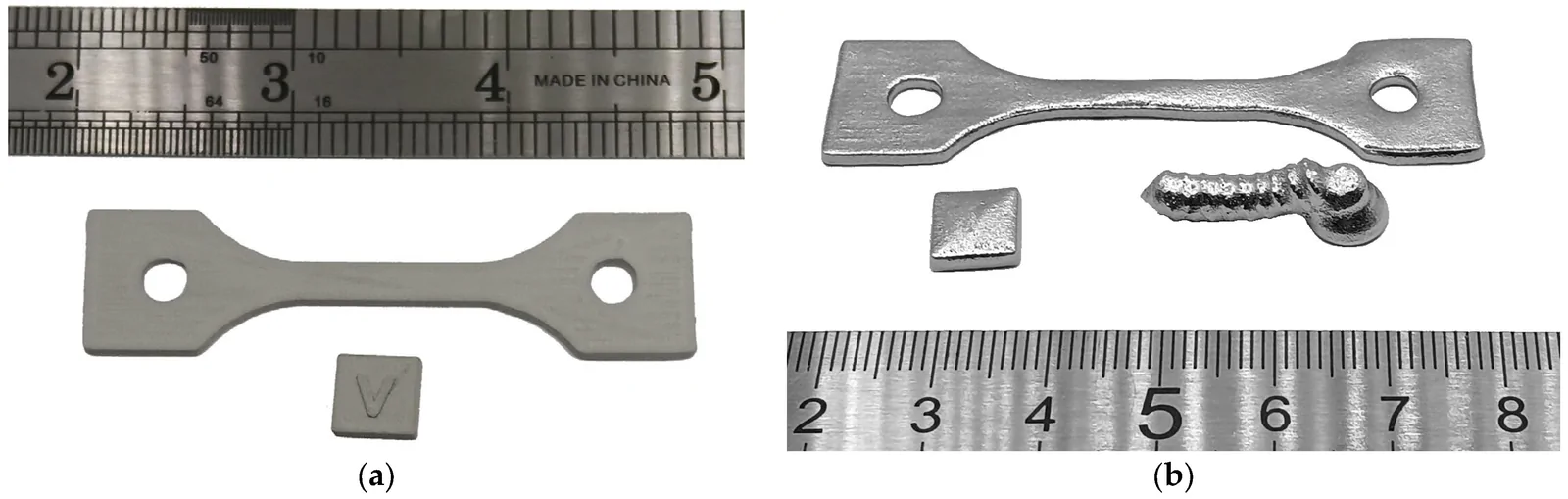
Sintered parts: (**a**) tensile test specimen (printed at 190 °C, 0.8 mm nozzle) and cuboid (printed at 190 °C, 0.4 mm nozzle) sintered at 1350 °C for 2 h (measuring bar in inch); (**b**) partially molten parts (tensile test specimen, cuboid, bone screw) at a sinter temperature of 1400 °C.

To investigate the influence of the sintering conditions on the final part quality, different sintering parameters were evaluated (Table 8). Unfortunately, the vendor of the alloy used did not supply any information on the melting temperature [33]. According to Patel, the ASTM F75 CoCrMo alloy possesses a melting temperature range, depending on the nitrogen and oxygen content, between 1377 and 1402 °C [38]. These values agree with other data reported in the literature with a melting range of 1379–1416 °C [39]. Fleming et al. reported a strong influence from the nitrogen and oxygen content on the melting behavior and found a broad range from 1484 up to 1571 °C [40]. Table 9 presents the obtained relative sinter densities as a function of the sinter parameters and printed samples. An influence from the printing nozzle on the sinter values could not be observed. For example, for the tensile test specimen in data set 3, the use of different nozzle diameters (0.4, 0.6, and 0.8 mm) yielded almost identical densities (93.3 ± 0.2%; 93.6 ± 0.3%; 93.8 ± 0.4%). In the following, all densities obtained irrespective of the print nozzle used were combined into a single mean value. Figure 9a shows a sintered tensile test bar as well as a cuboid. Both parts exhibit sharp outer contours. The temperature rise from 1350 to 1400 °C yielded a pronounced density increase to very high values around 99%. A closer look at the part appearance showed that partial melting had occurred (Figure 9b). Therefore, the maximum sinter temperature of 1350 °C delivered only poor density values around 94%, which is relatively low in contrast to, e.g., MF^3^-printed Ti6A4V parts [31,32]. De la Cruz sintered MEX-printed CoCrMo samples at 1375 °C for 2 h under vacuum and obtained density values between 96 and 98.5% [30]. Nevertheless, SLM-manufactured samples showed, e.g., density values between 91 and 97% th. [18].

#### 3.6.2. Shrinkage

**Figure 10:**
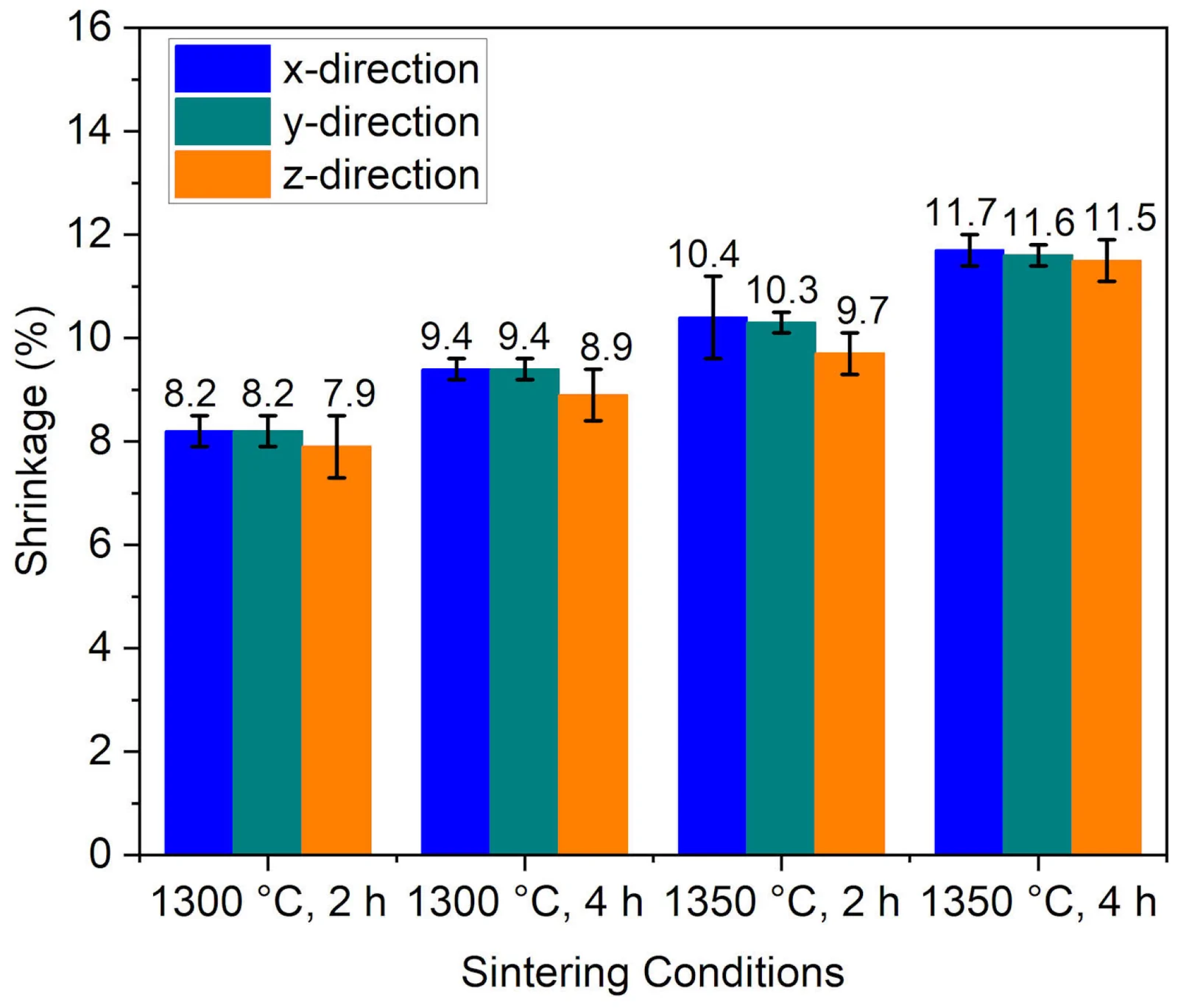
Measured shrinkage of the sintered cuboids.

Knowledge about the sinter shrinkage is important for future part design and overmeasure planning. According to the initial solid load of 60 Vol% in the feedstocks, a linear shrinkage of 15.7% can be estimated if an isotropic behavior is assumed. Figure 10 shows examples for the cuboids and different sinter parameter data sets with the measured linear shrinkage along the three axes as defined in Figure 2b. For all data sets, eight individual samples were considered; the given values are average values. The measured standard deviations are very small below ±0.8%.

Within the experimental error, the shrinkage in the x and y directions is isotropic, in the z direction, the values are a little bit smaller. The samples sintered at 1300 °C (2 h, 4 h) yielded small shrinkage values far away from the theoretical value of 15.7%. Sintering at 1350 °C (2 h, 4 h) led to a further increase in shrinkage but still away from the theoretical value. The relatively small sample shrinkage is in general accordance with the low sinter density data obtained that are listed in Table 9. Due to the loss of sharp outer contours, samples related to data set 5 were not measured.

#### 3.6.3. Nondestructive CT-Investigation

**Figure 11:**
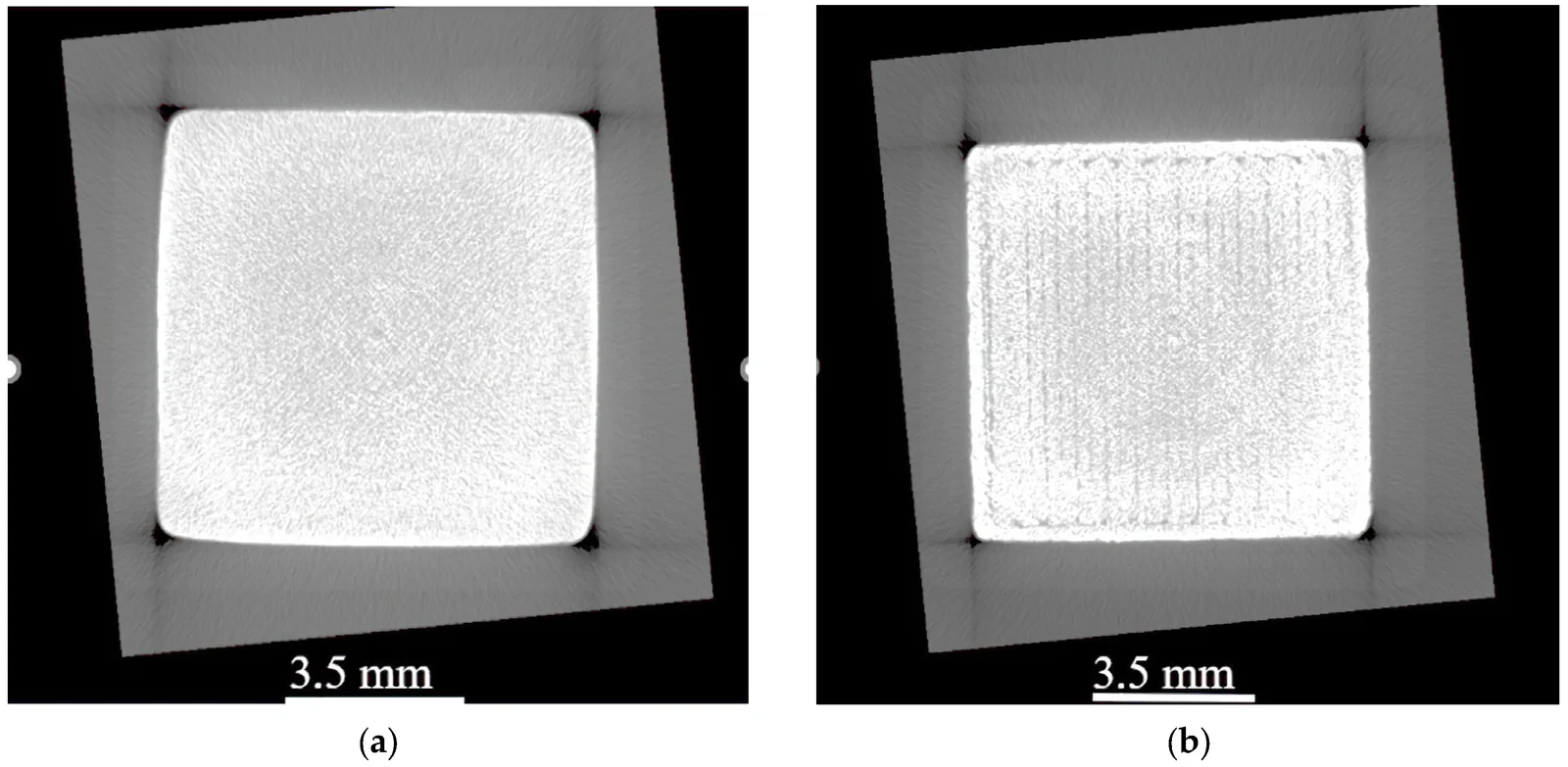
CT-images of two samples sintered (1350 °C, 4 h): (**a**) sample with 91.1% sinter density; (**b**) sample with 90.4% sinter density.

**Figure 12:**
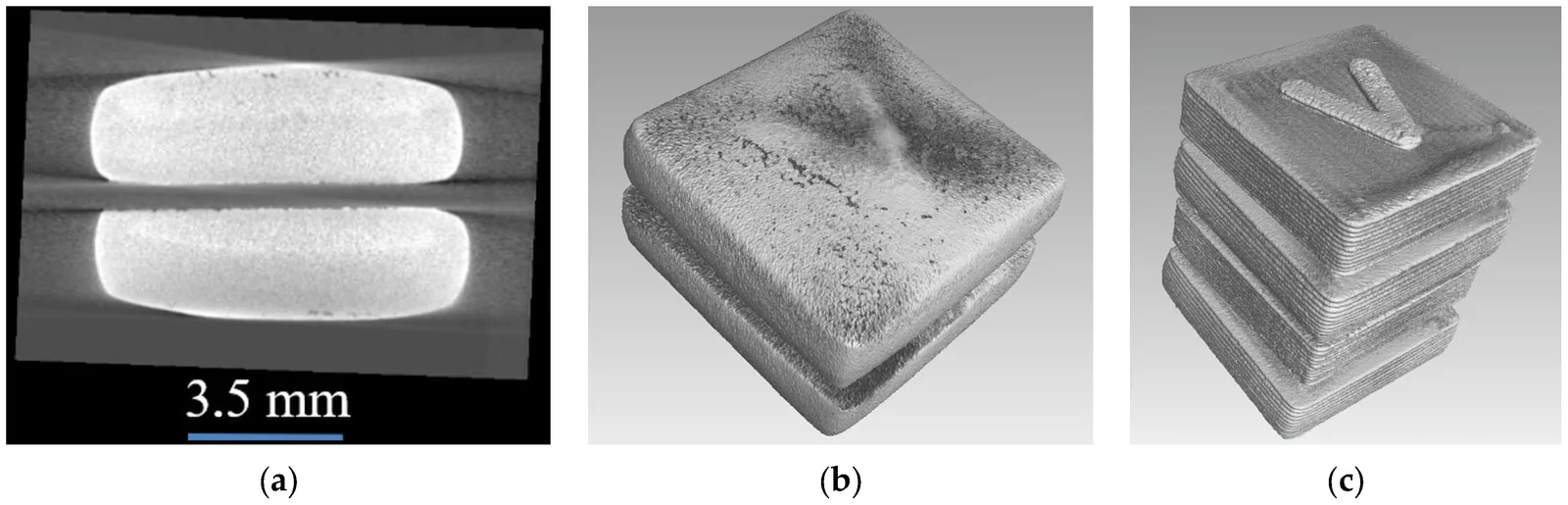
CT-scans of cuboids sintered at different temperatures: (**a**) 1400 °C, 2 h, cross section; (**b**) 1400 °C, 2 h, surface; (**c**) 1300 °C, 2 h (upper two parts), 4 h (lower two parts), surface.

The quality of the sintered parts was investigated by non-destructive CT-scans (Figure 11). CT has been selected to gain information if defects like voids are present in the bulk sample. This would help to understand measured density values and mechanical properties deviating from the literature or reference materials. Figure 11 presents two sintered samples from data set 4 (1350 °C, 4 h). The sample presented in Figure 11a possesses a relative density 91.1%, the one in Figure 11b of 90.4%. In the latter case, the individual deposited filament strands as well as a poor overlap of the infill with the wall can be seen. The sample in Figure 11a shows no defects above the CT’s spatial resolution of 15 µm. But according to the low density, a certain number of defects with sizes below 15 µm must be present. The partial melting of samples sintered at 1400 °C (2 h) can be clearly seen in Figure 12. Figure 12a shows the CT-scan of two samples (opposite faced). The upper surface in the oven exhibits a pronounced surface curvature, which is a strong hint for surface melting. Close to the upper surfaces, voids are present in the samples, which are visible in the surface visualization in Figure 12b. In contrast, a sinter temperature of 1300 °C (2 h, 4 h) presents sharp edges at the corners and the arrow structure as well as the outer contour of the z-axis with the visibility of individually printed layers (Figure 12c). These layer structures disappeared completely after melting (Figure 12b).

#### 3.6.4. Metallography

**Figure 13:**
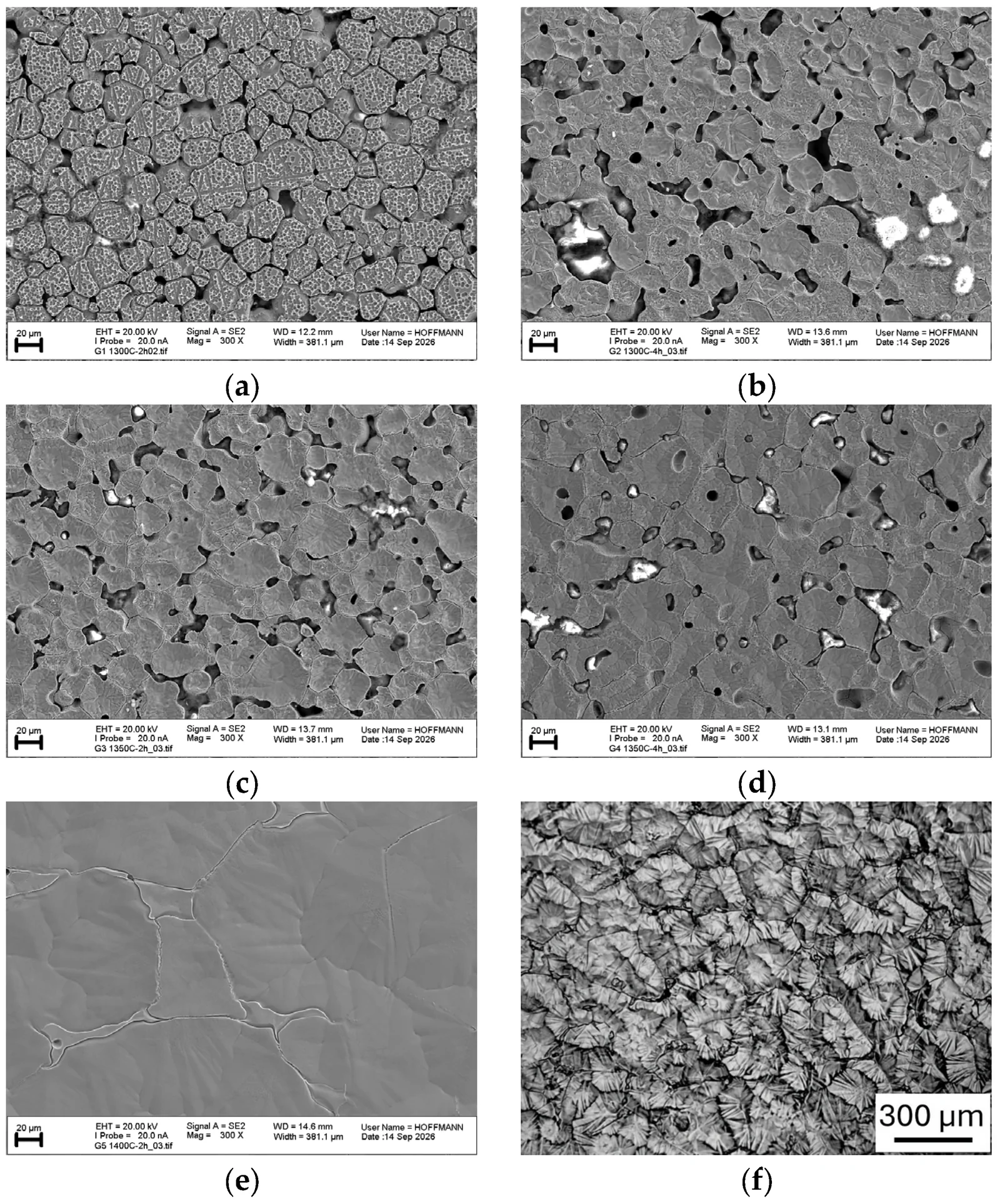
SEM images of surface appearances of sintered samples: (**a**) 1300 °C 2 h; (**b**) 1300 °C, 4 h; (**c**) 1350 °C, 2 h; (**d**) 1350 °C, 4 h; (**e**) 1400 °C, 2 h; (**f**) 1400 °C, 2 h, light microscopic image (bar length 300 µm).

Figure 13 visualizes the cuboid sinter progress with temperature and dwell time. In all cases the cross section was treated as described in Section 2.7. Sintering at 1300 °C for 2 h clearly shows the individual filament strands in a bundle-like shape (Figure 13a).

The dwell time extension to 4 h caused a converging of the strands by sinter neck formation (Figure 13b). At 1300 °C a huge number of voids can be detected, which explains the poor sinter density values (Table 9) around 92% th. The temperature rise up to 1350 °C delivers a clear sinter progress, especially at the 4 h dwell time, losing the pristine filament appearance (Figure 13d). Still a huge number of voids can be seen, but the voids possess a smaller size, and grain growth is obvious. The final temperature increase to 1400 °C yielded a pronounced grain growth and a complete void disappearance in good agreement (Figure 13e) with the measured density around 99% th. A light microscope image of the same sample with course optical magnification factor shows that the grains seem to have rounded edges, which can be attributed to previously reported local melting (Figure 13f). The average grain size is around 66.3 µm. A similar texture of CoCrMo F75 was obtained by Haan et al. applying selective laser melting [41]. The specimen showed a density around 99.7% th., which agrees with the value described in this work. A similar texture was found by Sing et al. as well [17].

#### 3.6.5. Mechanical Properties

**Figure 14:**
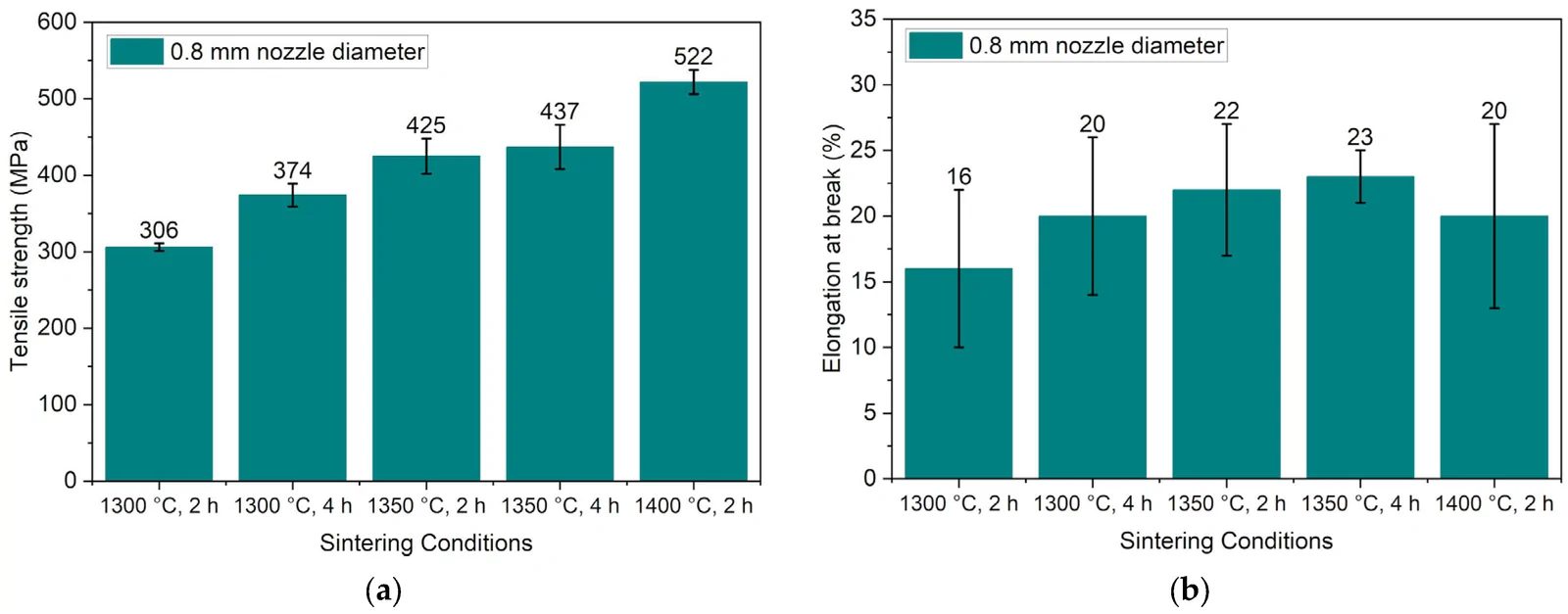
Influence of sinter conditions on mechanical properties (nozzle diameter 0.8 mm): (**a**) tensile strength; (**b**) elongation at break.

With respect to application, e.g., as implants, knowledge about the mechanical properties of a sintered material is mandatory. Figure 14a presents the tensile strength of a sintered tensile test specimen (Figure 2a), printed with a nozzle diameter of 0.8 mm applying different sinter conditions (Table 9). For each data set, five samples were investigated. In agreement with the measured densities (Table 9) and more pronounced with the shrinkage values (Figure 10), increasing sinter temperatures and dwell times yielded higher tensile strengths. As described earlier, the use of data set 5 (1400 °C, 2 h) caused a partial melting of the sample, minimizing bulk and surface defects generating higher tensile strength values. The related elongation at break (EAB) data follows the same above-described trend (Figure 14b), only in the case of data set 5 can a slight decrease be observed.

This behavior may be attributed to the different textures as seen in Figure 13e,f. In the literature, a wide range of measured UTS and EAB can be found: Fleming et al. reported for investment casting (1470 °C) ASTM F75 CoCrMo samples, ultimate tensile strength values around 847.5 MPa and an EAB of 12% [40]. Zhuang and Langer found for as cast F75 CoCrMo samples UTS values of 655 MPa and EAB values of 8.0% [12]. Dan et al. reported for investment casted test samples UTS-values of 562 MPa and 6.1% elongation [13]. A tensile test specimen, fabricated by selected laser melting (SLM), showed a UTS-value of 1070 MPa as built [17]. Unfortunately, the authors did not measure the local melt temperature during processing. The final mechanical properties can be significantly influenced by thermal treatment at different temperatures after casting [13]. The formation of carbides yielded a change in the UTS and elongation values: the higher the temperature, the lower the final UTS value [13]. To sum up, the results given for the UTS are smaller than the data found in the literature. May be one reason can be the surface roughness as detected e.g., in Figure 11b originating from the individual filament strands. But the EAB values are significantly higher than the data reported in the literature.

### 3.7. Current Process Limitation

One of the most critical steps is sintering. An ASTM F75 CoCrMo alloy exhibits, depending on the exact composition and possible impurities, a melting range from approximately 1330 to 1410 °C. The related published work from Garcia de la Cruz applying FGF used a sinter temperature of 1375 °C [30], Pellegrini et al. with binder jetting as shaping method applied 1325 °C as maximum sinter temperature [29]. To achieve high density values under retention of the structural accuracy, melting must be strictly avoided. Vendors’ materials data sheets do not list a melting temperature normally. Hence the real melting behavior must be evaluated by dilatometry and differential scanning calorimetry in advance for sinter-parameter evaluation. Other critical process limitations derive from the MF^3^ process chain, like maximum solid load and printability. Due to the very small numbers of published works applying MEX as additive manufacturing method for CoCrMo alloys, a general statement on the process limitations cannot be given.

### 3.8. Relevance of MEX Methods for the Realization of CoCrMn Parts

According to the state of the art described in the literature, the presented work is the first focusing on FFF in addition to the groundbreaking work of Garcia de la Cruz covering the FGF technology [30]. MEX methods like MF^3^ possess in contrast to SLM and EBM two main advantages: first, the resulting metal morphology is isotropic, and second, the investment costs are significantly lower. In the case of a modern EBM machine including peripheral equipment and infrastructure installation a total investment up to EUR 3 million can be assumed [42]. The investment for SLM is lower but including peripheral equipment it can rise to EUR 1.5 million [43]. The investment for production machines are significantly higher. In both cases, security issues due to the large amount of fine metal powder are pronounced cost drivers. In contrast, a modern and reliable FFF printer is achievable below EUR 2000. With respect to the necessary whole process chain, starting with compounding via printing and ending with a suitable furnace, which can be driven under inert gas or vacuum, the total investment costs can be around EUR 300,000. Hence FFF or better MF^3^ can contribute to a significant cost reduction. But as a prerequisite, the whole process chain must achieve a higher TRL, which can be achieved by further research efforts. A very detailed comparison between MF^3^ and laser powder bed fusion was undertaken by Parenti and coworkers [44]. They found, depending on the number of produced parts and the selected machine, a lower cost per part for those fabricated via MF^3^ [44].

## 4. Conclusions

In this research, new feedstock compositions consisting of 60 Vol% CoCrMo F75-23 powder and partially water-soluble PEG/PMMA binders with ATBC as plasticizer were developed and successfully tested for use in the MF^3^ process chain. The most important results are concluded in the following:

1. The presence of 10 Wt% ATBC allowed for good compounding behavior, acceptable melt viscosity, reliable filament fabrication by extrusion and enabled for an almost defect-free FFF printing without grinding and filament breakage.
2. A set of printing parameters was found by applying different extrusion nozzles.
3. The essential parameters for the three-step debinding process were evaluated.
4. A careful sinter temperature adjustment must be performed to avoid sample melting.
5. The obtained sintered densities were lower than those achieved by conventional manufacturing such as casting.
6. The measured tensile strength values were lower than data reported in the literature.
7. The measured elongation at break values were comparable to or higher than values reported in the literature.

From the results obtained, a first set of parameters can be given as a starting point for further investigations, e.g., described in the outlook section. The binder composition ATBC 10.0 with a filler load of 60 Vol% was the only one which could be printed in a reliable manner. A set of printing parameters was found after optimization with respect to print quality, especially since the huge printing temperature of 190 °C delivered best results. Debinding and sintering follows established or common parameters. A very precise temperature control during sintering passing 1350 °C is mandatory to avoid alloy melting.

## 5. Outlook

The promising results obtained show still the clear need for further material and process optimization targeting higher sinter densities accompanied by an improvement in the mechanical properties. One central step can be the increase in the solid load to 65 Vol% together with an optional adjustment of the binder composition if necessary. In addition, a closer sintering temperature like 1375 °C towards the melting temperature for enhanced properties should be undertaken.

## Figures and Tables

**Table 1:** Selected measured particle properties.

Material	Density (g/cm^3^)	d_10_ (µm)	d_50_ (µm)	d_90_ (µm)	Specific Surface Area (m^2^/g)
CoCrMo	8.38	19.2	27.6	39.5	0.15

**Table 2:** Binder components used, their functions, and the related suppliers.

Binder Component	Function	Supplier
PMMA (Degalan G7E)	Backbone polymer	Röhm GmbH, Darmstadt, Germany
PEG 8000	Main component	C. Roth GmbH + Co. KG, Karlsruhe, Germany
ATBC	Plasticizer	C. Roth GmbH + Co. KG, Karlsruhe, Germany

**Table 3:** Binder compositions investigated. 100 Vol% binder equals 40 Vol% in the feedstock at a constant solid CoCrMo load of 60 Vol%.

Denomination	ATBC (Wt%)	ATBC (Vol%)	PMMA (Vol%)	PEG (Vol%)
ATBC 7.5	7.5	8.6	40	51.4
ATBC 10.0	10.0	11.3	40	48.7
ATBC 12.5	12.5	14.1	40	45.9

**Table 4:** Temperature program selected for thermal pre-debinding necessary for ATBC removal.

Temperature Range (°C)	Heating/Cooling Rate (°C/min)	Dwell Time (min)
25–120	0.2	-
120	-	30
120–180	0.2	-
180	-	60
180–230	0.2	-
230	-	120
230–25	1	-

**Table 5:** Combined temperature program for thermal debinding and sintering.

Temperature Range (°C)	Heating/Cooling Rate (°C/min)	Dwell Time (min)
25–550	1.5	-
550	-	60
550–(1300, 1350, 1400)	5	-
1300, 1350, 1400	-	120, 240
(1300, 1350, 1400)–25	15	-

**Table 6:** Optimized extrusion temperatures and filament diameter for all CoCrMo feedstocks.

Feedstock	Extrusion Temperature (°C)	Filament Diameter (mm)
ATBC 7.5	70	2.80 ± 0 04
ATBC 10.0	80	2.86 ± 0.03
ATBC 12.5	80	2.83 ± 0.02

**Table 7:** Applied printing parameters.

Parameter	Value
Temperature (°C)	180–190
Nozzle diameter (mm)	0.4/0.6/0.8
Built plate temperature (°C)	30–40
Layer thickness (mm)	0.1 (first layer), 0.2 (other layers)
Infill (%)	105
Overlap infill with wall (mm)	0.4–0.6
Speed (mm/s)	8

**Table 8:** Denomination and related sinter parameters and number of samples investigated.

Data Set	Max. Temperature (°C)	Dwell Time (h)	Number of Cuboids	Number of Tensile Test Specimen
1	1300	2	8	5
2	1300	4	8	5
3	1350	2	8	15
4	1350	4	8	15
5	1400	2	8	5

**Table 9:** Measured relative density values as function of the applied sinter parameters.

Data Set	Temperature (°C)	Dwell Time (h)	Cuboid Sinter Density (%)	Tensile Test Specimen Sinter Density (%)
1	1300	2	91.8 ± 3.7	94.3 ± 0.3
2	1300	4	91.7 ± 6.0	94.7 ± 0.3
3	1350	2	93.5 ± 0.8	93.7 ± 1.1
4	1350	4	91.1 ± 1.9	93.7 ± 0.6
5	1400	2	99.3 ± 0.3	99.6 ± 0.2

## Data Availability

The original data presented in the study are openly available in KITOpen Repository, DOI: 10.35097/wnsckackrnxke6kf.

## Abbreviations

The following abbreviations are used in this manuscript:

AM	Additive manufacturing
ASTM	Advancing Standards. Transforming Markets (pristine name: American Society for Testing and Materials)
ASTM F136-26	Standard specification for wrought Titanium-6Aluminum-4Vanadium ELI (extra low interstitial) alloy for surgical implant applications
ASTM F1537-20	Standard specification for wrought Cobalt-28Chromium-6Molybdenum Alloys for surgical implants
ASTM F2885-17	Standard specification for metal injection molded Titanium-6Aluminum-4Vanadium components for surgical implant applications
ASTM F75-23	Standard specification for Cobalt-28 Chromium-6 Molybdenum alloy castings and casting alloy for surgical implants
ASTM F799-19	Standard specification for Cobalt-28 Chromium-6 Molybdenum alloy forgings for surgical implants
ATBC	Acetyltributylcitrate
BET	Brunauer–Emmett–Teller
CF^3^	Ceramic fused filament fabrication
CoCrMo	For medical implants used alloy with major elements mentioned
CT	Computed tomography
EAB	Elongation at break
EBM	Electron beam melting
FFF	Fused filament fabrication
HDPE	High-density polyethylene
LDPE	Low-density polyethylene
MEX	Material extrusion
MF^3^	Metal fused filament fabrication
MIM	Metal injection molding
PEG	Polyethylene glycol
PEG 8000	Polyethylene glycol with an average mass of 8000 g/mol
PMMA	Polymethylmethacrylate
SA	Stearic acid
SLM	Selective laser melting
Ti-6Al-4V (Ti64)	For medical implants used alloy with major elements mentioned
TTB	Tensile test bar
UTS	Ultimate tensile strength
Vol%	Volume%
Wt%	Weight%
